# An Analysis of the Esthetic Proportions of Anterior Maxillary Teeth in a School of Dentistry in Ar Rass

**DOI:** 10.7759/cureus.51040

**Published:** 2023-12-24

**Authors:** Moustafa Omran, Hammad Alshyai

**Affiliations:** 1 Prosthodontics, Qassim University, Al Rass, SAU; 2 Dentistry, Qassim University, Al Rass, SAU

**Keywords:** recurrent esthetic dimensions, dental esthetic, golden percentages, golden proportion, esthetic proportions

## Abstract

Background: There are several facets of esthetic dentistry, such as the shade and color of teeth, but the geometric or mathematical proportion aspect is among the most crucial. Theories such as the golden percentage, golden proportion, and recurring esthetic dental are presented in esthetic dentistry.

Material and methods: The present prospective observational research was undertaken between January 2022 and January 2023. The volunteers’ cohort included individuals aged 18 to 45 years without periodontal disease, free of carious cavities and restorations in the anterior teeth, with a complete set of anterior teeth and at least one quadrant of well-aligned anterior maxillary teeth. We utilized an iPhone mobile lens (Apple Inc., Cupertino, CA) to take photos. The maximum circumference on the mesial and distal contacts of anterior teeth was used as a reference point for measurements. After data collection, a customized Excel spreadsheet (Microsoft Corporation, Redmond, WA) was formulated to facilitate mathematical analysis.

Results: This study was conducted on 60 dental students, 43 being male subjects and 17 female subjects. The best results in this study were seen in relation to perceived central incisor width and perceived lateral width as seen from the front. It was found that the unidentified group appears to be the most relevant group with 33 participants (52%), and Preston’s proportion was the least frequent with 0 participants (0%). As expected, males appeared to have larger anterior teeth dimensions than females.

Conclusions: The actual correlation between maxillary teeth width as perceived from the front view is unidentified. Golden percentage and modified Golden percentage ranked second and third. Preston’s proportion did not exist. Based on our study, a new formula for calculating the relation between maxillary anterior teeth width could be raised and should be tested on a wider population.

## Introduction

There are several facets of esthetic dentistry, such as the shade and color of teeth, but the geometric or mathematical proportion aspect is among the most crucial. Theories such as the golden percentage, golden proportion, and recurring esthetic dental are presented in esthetic dentistry [1-4].

The use of the golden proportion in dentistry was initially proposed by Lombardi [5]. Continuing Lombardi's work, Levin [6] proposed determining relative tooth size from a frontal view by applying the golden proportion. He suggested that the perceived width of the maxillary central incisor should be in golden proportion to the width of the lateral incisor (1:0.618) and that the width of the lateral incisor should also be in golden proportion to the perceived width of the canine. Levin also adds that efforts to establish a connection between the incisors' actual measured widths have proven unsuccessful. Moreover, Preston [7] discovered that the connection between the maxillary central incisor and the mandibular lateral incisor does not follow the golden proportion of 1.618:l.

The golden percentage was introduced by Snow [8] in 1999, as a more precise technique for establishing symmetry, dominance, and proportion in smiles that are esthetically pleasant. He used the idea of the golden proportion as a basis for his calculation and derivation of this golden percentage. Snow used the golden proportion ratio of 1.618 for the central incisor, 1 for the lateral incisor, and 0.618 for the canine and multiplied each of the numbers by two to account for both sides of the arch. Upon obtaining the total of the six values, 6.472, he proceeded to divide each tooth ratio by the overall ratio to determine the golden percentage. Snow's golden percentage showed that the entire width of anterior teeth on one side should be 25% for the central, 15% for the laterals, and 10% for the canines for an esthetically pleasing smile.

A different esthetic proportion called recurring esthetic dental (RED) proportion was proposed by Ward [9] in 2001. It was based on Lombardi's theory of using repeating proportionate widths. He believed that the golden proportion makes the lateral incisor appear too narrow and reduces the display of the canine. He proposed that as one proceeds distally, the ratio of the successive widths of the teeth, as seen from the frontal perspective, should stay constant. However, rather than being locked with the 62% proportion, the dentist is free to choose any proportion they like as long as it remains constant while moving from the central incisor to the canine.

Studies reported that Preston proportion or modified golden percentage improves proportion. Maxillary central incisors have a width-to-height ratio of 0.75-0.85 [10,11]. Ahmed et al. [12] reported that the RED proportions are not the sole norm for restoring attractive smiles across the world, because anterior tooth proportions vary by race and ethnicity.

Based on a study on Saudi people, in the capital city, conducted by Aldegheishem et al. [13], no gold standard was identified. In addition to the measures of the anterior teeth, esthetic evaluations should consider unique demographic features and the sense of a pleasing smile. This observation was validated in Turkish individuals, whose maxillary front tooth proportions depart considerably from both the golden proportion and the recurring esthetic dental proportion [14]. However, slightly adjusted golden percentage values are highly relevant and are suggested as a more pertinent geometric component to esthetic principles in the United Kingdom [15]. Wu et al. [16] have observed that individuals in China have a different width/height ratio of the clinical crown and varied than those of people in other countries.

Researchers in Spain used smile images from the general population to conclude that only modified golden proportion values with a variation of 1% are primarily appropriate [17]. In a more comprehensive study, Akl et al. [18] clarified that the mathematical calculations were not reliable enough to be used as a universal guide and that the actual mathematical formula for any population may be governed by several characteristics.

The aim of the current study is to analyze maxillary anterior teeth proportions from dental students of Ar Rass Dental College and compare the categorized groups with the current theories of esthetic proportions to be a base for wider sample groups and parameters. The data were also statistically analyzed to find a suitable pattern or correlation for the current sample studied. The alternative hypothesis was that the present findings match one of the well-established proportions for the esthetic proportion of maxillary front teeth.

## Materials and methods

Study design

The present prospective observational research was undertaken between January 2022 and January 2023. The study adhered to the research ethical norms and regulations of the research bioethics that govern research in Saudi Arabia. The study was approved by the Ethical Committee of Research Ethics, Deanship of Scientific Research, Qassim University (registration number: 21-12-01). After simple random sampling, 75 individuals from Ar Rass Dental College were assessed for eligibility using the predetermined inclusion criteria. Accordingly, 60 individuals were enrolled in the study.

The participants' cohort included individuals aged 18 to 45 years. The inclusion criteria were participants without any systemic disease, free of periodontal disease, free of carious cavities and restorations in the anterior teeth, with a complete set of anterior teeth and at least one quadrant of well-aligned anterior maxillary teeth. The exclusion criteria were participants who had systemic disease, periodontal disease, carious cavities, or restorations in the anterior teeth and missing anterior teeth (Table 1).

**Table 1:** Inclusion and exclusion criteria.

Inclusion criteria	Exclusion criteria
Participants without any systemic disease	Participants who had systemic disease
Free of periodontal disease	Have periodontal disease
Free of carious cavities and restorations in the anterior teeth	Have carious cavities or restorations in the anterior teeth
With a complete set of anterior teeth and at least one quadrant of well-aligned anterior maxillary teeth	Have missing anterior teeth

Photo capturing protocol

The photos were acquired using the "whole smile frontal view, 1:2 magnification, and non-retracted view," as recommended by the "American Academy of Cosmetic Dentistry" (AACD) [17]. One photographer (H.S.) took all the shots, and they were all taken in the same conditions with the subjects in the same lighting in a calibrated condition. Each participant's head was placed on a Frankfort plane that was perpendicular to the floor. We utilized an iPhone mobile lens (Apple Inc., Cupertino, CA) in professional mode to have full control of the camera parameters. Camera specifications were 12MP camera system (ultra-wide, wide, and telephoto) modes. The ISO was set at 200 to avoid noise, the shutter speed was set to 1/125 to get a sharp image, the aperture diaphragm opened to f/20 to keep all maxillary anterior teeth in focus, and the distance to the subject was set to 0.49 m to avoid distortion. The magnification ratio was set to 1:2.

**Figure 1:**
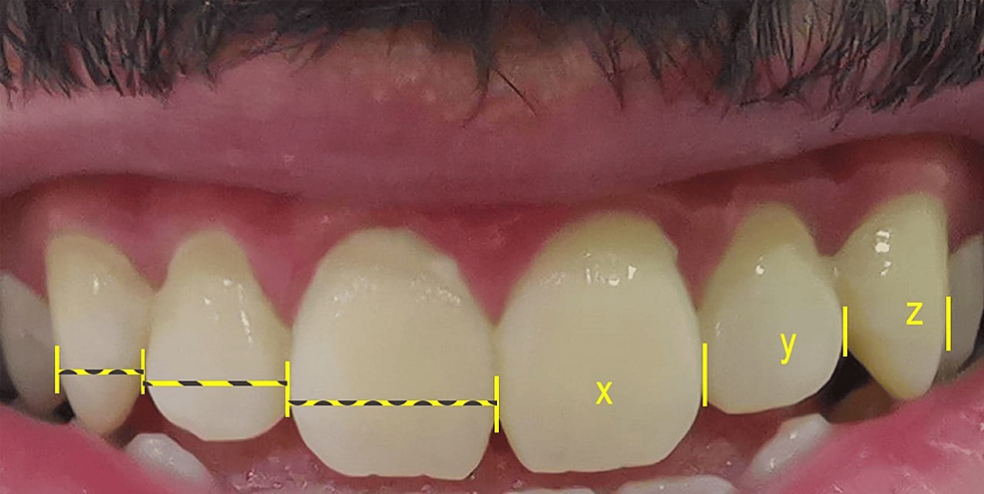
Representative image after using the ruler to measure the distance between the reference points. Where X, Y, and Z are the width of the central incisor, lateral incisor, and canine tooth, respectively.

All photos were checked for their quality and suitability for enrolment and the image was excluded and repeated for any deviation from the required criteria. An Apple application was installed and used (Photo Meter -Picture Measuring). The maximum circumference on the mesial and distal contacts of anterior teeth was used as a reference point for measurements. The application enables the use of the ruler after calibration to measure the distance between the reference points and save the data for further export (Figure 1). Measurements of the teeth were then exported in an Excel spreadsheet (Microsoft Corporation, Redmond, WA) to be ready for analysis.

Statistics

After data collection, a customized Excel spreadsheet was formulated to facilitate mathematical analysis, where the data were compared to the different esthetic concepts (G Prop = golden proportion, RED Pro = recurrent esthetic dental proportions, G% = golden percentage, and MG% = modified golden percentage) using pre-customized Excel equations. If the data did not match any of these concepts, they were counted as an unidentified correlation (Table 2). In addition, statistical analyses of the data collected were calculated by statistical analysis software (IBM SPSS Statistics, v21.0; IBM Corp., Armonk, NY) to show descriptive statistics and correlations that may be found in the unidentified group. The proper correlation test was selected after testing data normality.

**Table 2:** Pre-customized Excel sheet where data were compared with the well-known esthetic proportion. Cen: central incisor; Lat: lateral incisor; Can: canine; W: width; G Pop: golden proportion; RED Prop: recurring esthetic dental proportion; Prens Prop: Preston's proportion; G%: golden percentage; MG%: modified golden percentage; Unident: unidentified.

Number	Gender	W cen	W lat	W can	v Ce_La	v Lat_Can	Cen %	Lat %	Can %	G Prop	RED Prop	Prens Prop	G%	Unident
41	M	8	5	3	0.63	0.60	25.00	15.63	9.38	Yes	No	No	Yes	-
42	M	8.5	4	3.5	0.00	11247.50	25.04	0.00	24.96	No	No	No	No	No
43	M	6.5	4.5	3.5	1.00	1.00	16.69	16.66	16.65	No	Yes	No	No	-
44	M	7	5	4.5	0.71	9004.20	0.01	0.01	49.99	No	No	No	No	No
45	M	6.5	4.5	3.5	1.00	1.00	16.69	16.66	16.65	No	Yes	No	No	-
46	M	7	3	4	0.43	1.33	25.00	10.71	14.29	No	No	No	No	No
47	M	6.5	3.5	2.5	1.00	1.00	16.69	16.66	16.65	No	Yes	No	No	-
48	M	7.5	3.5	2.5	1.00	1.00	16.70	16.66	16.64	No	Yes	No	No	-
49	M	7	3.5	2.5	6427.14	1.00	0.00	25.01	24.99	No	No	No	No	No
50	M	8	6	4	0.75	0.67	22.22	16.67	11.11	No	No	No	No	No
51	M	7.5	4.6	3	1.00	0.00	25.02	24.97	0.00	No	No	No	No	No
52	M	7	4.5	3.5	6431.57	1.00	0.00	25.01	24.99	No	No	No	No	No
53	M	6	4	3.5	0.67	11247.50	0.01	0.00	49.99	No	No	No	No	No
54	M	6.5	4	3	0.00	0.75	49.99	0.00	0.00	No	No	No	No	No
55	M	7.5	4.5	3.5	1.00	1.00	16.69	16.66	16.65	No	Yes	No	No	-
56	M	6	3.5	3	7498.33	0.00	0.01	49.99	0.00	No	No	No	No	No
57	M	6	5	3	0.83	0.60	21.43	17.86	10.71	No	No	No	No	No
58	M	6	3.5	3.5	7498.33	1.00	0.00	25.00	25.00	No	No	No	No	No
59	M	6	4.5	3.5	7503.50	1.00	0.00	25.01	24.99	No	No	No	No	No
60	M	8.5	5	3	0.00	0.60	49.99	0.01	0.00	No	No	No	No	No

## Results

**Figure 2:**
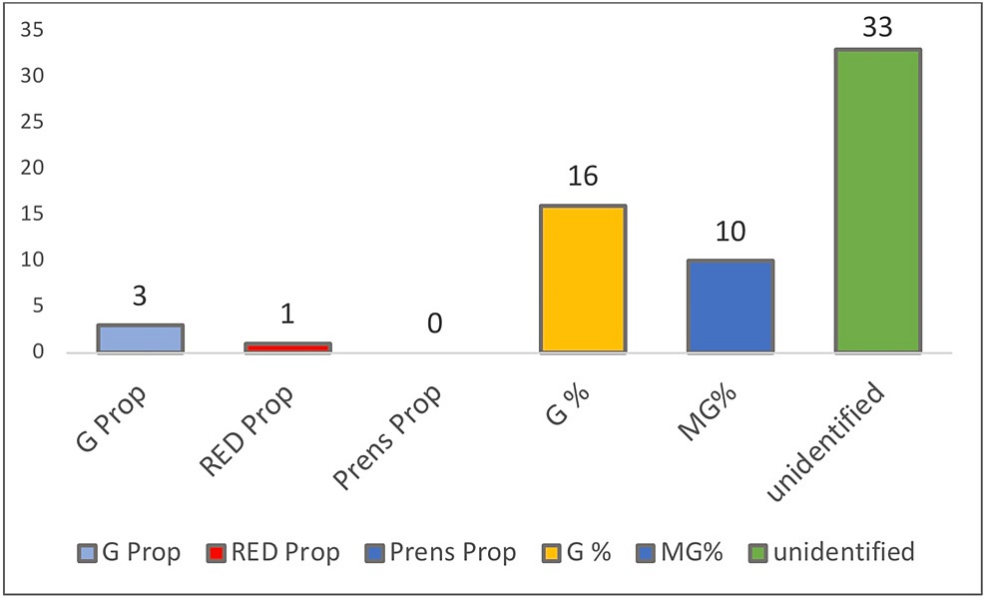
Frequency of incidence of each esthetic concept in all the participants. G Prop: golden proportion; RED Prop: recurring esthetic dental proportion; Prens Prop: Preston’s proportion; G%: golden percentage; MG%: modified golden percentage.

**Figure 3:**
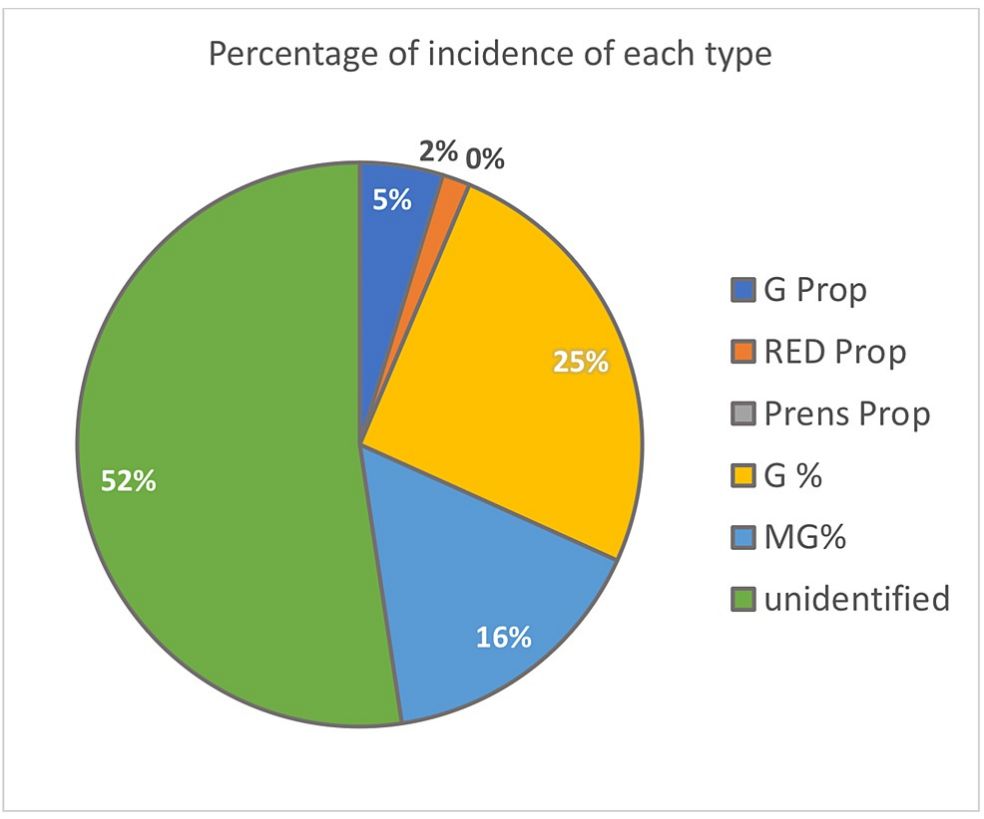
The percentage of incidence of each esthetic concept for all the participants. RED Prop: recurring esthetic dental proportion; Prens Prop: Preston’s proportion; G%: golden percentage; MG%: modified golden percentage.

The unidentified group was the most relevant among the others with 33 participants, which is 52%, whereas the golden percentage was the second with 16 participants (25%), and Preston’s proportion was the least frequent with 0 participants (0%), as seen in Figures 2, 3.

In male participants, the unidentified group was the most relevant with 27 participants whereas in females, it was the modified golden percentage with nine participants (as seen in Table 3).

**Table 3:** Segregation of the data based on the participant’s gender. G Pop: golden proportion; RED Prop: recurring esthetic dental proportion; Prens Prop: Preston’s proportion; G%: golden percentage; MG%: modified golden percentage.

Gender	G Prop	RED Prop	Prens Prop	G%	MG%	Unidentified
Male	3	1	0	14	10	27
Female	0	0	0	2	9	6
Total	3	1	0	16	10	33

The Kolmogorov-Smirnov test was chosen to examine the normality of the data. After checking the data for normalcy, non-parametric data were discovered. In this manner, Spearman's test was chosen to calculate the correlation between teeth widths (Table 4).

**Table 4:** Normality testing of the data using the Kolmogorov-Smirnov test.

Tooth	Test statistic	Number of samples (df)	Level of significance (p-value)	˂0.05
Central incisor	0.140	60	0.005	Significant
Lateral incisor	0.141	60	0.005	Significant
Canine	0.208	60	0.000	Significant

The Kolmogorov-Smirnov test was carried out to determine whether all three variables, i.e., central incisor, lateral incisor, and canine, follow the normal distribution.

The results of the Kolmogorov-Smirnov test indicate that variables are not normally distributed as the p-value is significant, which suggests that all three variables do not follow a normal distribution.

**Figure 4:**
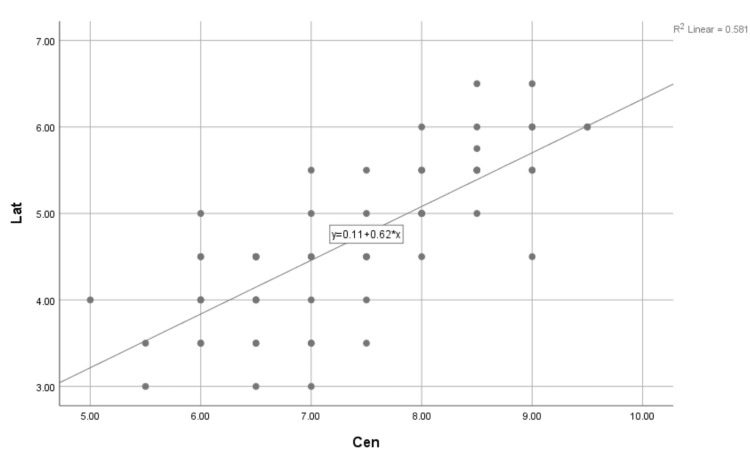
Nonparametric correlations of the data of lateral incisors and canines. Chart plotter showing the distribution of the data, correlation coefficient, and the correlation equation. X = lateral incisor; Y = central incisor.

The Spearman’s test showed a positive correlation between central incisors and lateral incisors with a significance <0.001 and a correlation coefficient of 0.765 (Table 5 and Figure 4).

**Table 5:** Nonparametric correlations of the data of central incisors and lateral incisors. ** Correlation is significant at the 0.01 level (two-tailed).

Number of samples (N)	Correlation coefficient	Level of significance (p) two-tailed
60	0.765^**^	˂0.001

**Figure 5:**
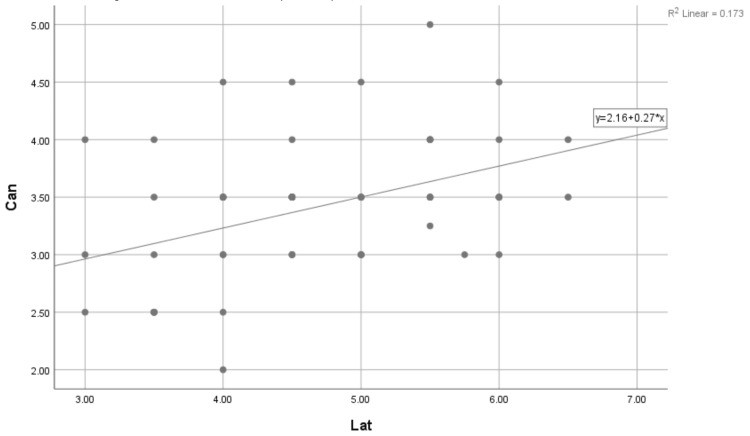
Nonparametric correlations of the data of lateral incisors and canines. Chart plotter showing the distribution of the data, correlation coefficient, and the correlation equation. X = lateral incisor; Y = central incisor.

The correlation coefficient of 0.765 suggests that there is a positive correlation between the central incisor and the lateral incisor. P < 0.001 indicates that the correlation between the two variables is highly significant. The relationship of the central incisor with lateral incisors as calculated was Y = 0.11 + 0.62 * x in R2 = 0.581. Similarly, the relation of the lateral incisors to canines after doing Spearman’s test was significant (p < 0.001), with a correlation coefficient of 0.411 (Table 6 and Figure 5).

**Table 6:** Nonparametric correlations of the data of lateral incisors and canine (Spearman’s test) ** Correlation is significant at the 0.01 level (two-tailed).

Number of samples (N)	Correlation coefficient	Level of significance (p) two-tailed
60	0.411^**^	˂0.001

The correlation coefficient of 0.411 suggests that there is a positive correlation between the lateral incisors and canines. P < 0.001 indicates that the correlation between the two variables is highly significant. The relationship of the lateral incisors with the canine was Y = 2.16 + 0.27 * x in R2 = 0.173.

## Discussion

This study aimed to analyze maxillary anterior teeth from Ar Rass Dental College and to achieve an esthetic restoration since it is crucial to ascertain a mathematical or geometrical relationship between the teeth. It would be beneficial if statistically valid relationships supported the present relationship hypotheses.

The results showed that the correlation between maxillary teeth width as perceived from the front view is unidentified for most of the study's cohort. The alternative hypothesis was thus rejected, and most individuals exhibited unidentifiable concepts.

This study was conducted on 60 dental students, 43 male and 17 female subjects. The best results in this study were seen with perceived central incisor width and perceived lateral width as seen from the front, as the correlation coefficient was 0.765. It was found that the unidentified group appears to be the most relevant group, with 33 participants (52%), and Preston's proportion was the least frequent, with 0 participants (0%).

In this study, the measurements were made on the photographs, a method also reported in Wu et al.’s study [16], where they used cast digital photographs for their analysis. Rodríguez-López et al. [17] also used smile photographs in their study.

The clinical crown dimensions for maxillary anterior teeth were ranked from largest to smallest as central incisor, lateral incisor, and canine, which agree with the findings of Özdemir et al. [14].

As expected, males appeared to have larger anterior teeth dimensions than females [9].

Based on the present study results, most of the participants did not show matching with the known concept. Accordingly, the correlation between teeth widths was calculated, and a new correlation was found, which was unidentified with 33 participants (52%).

This finding could be in harmony with Rodríguez-López et al. [17], who confirmed in their study that the golden proportion, RED, golden percentage, and Preston's percentage requirements are not met with a minor trend for new values to be referred to as modified golden percentages. Similar research demonstrated that the RED proportion idea, as applied to natural dentition, is unsupported by data. The RED percentage findings obtained were 2%, which were comparable to those of Murthy et al. [19], as the RED proportion was not found to exist between the six maxillary anterior teeth, and Shetty et al. [20], as the RED proportion was not seen in natural dentition.

On the other hand, the data suggested that the Preston percentage did not exist. This was consistent with the research of Ahmed et al. [12] and Akl et al. [18], who measured the mesiodistal breadth of six anterior teeth on scanned images of people to determine the existence of Preston's proportion.

The current study reported a more modified golden percentage in the female group, which was found in nine participants, while the unidentified category for most male groups, which was found in 27 participants. Similarly, Forster et al. [21] did not find statistically significant differences when comparing tooth width according to sex, although they did register higher values for men's teeth. Perceptual theories, which discourse dental esthetics based on how we "see" or perceive a beautiful smile, have recently gained popularity. Therefore, it may be fundamental and naive to analyze dental esthetics and base suggestions on a single factor, such as tooth proportion.

The primary drawback of our study was a smaller sample size of 60 participants, while studies by Forster et al. [21] used 101 participants, and Özdemir et al. [14] used 150 participants. Some variations in our measurements could be introduced due to the use of photographs taken with an iPhone camera. The digital caliper measurements and the tiny positioning deviations that can occur during photography may have also affected the findings. However, all measures were taken to ensure consistency in calibrated conditions. Nonetheless, the study contributed some valuable data about the size of the front teeth of Ar Rass Dental College students. It could be a good start for more insight into the wider population.

Further research on the dimensions of teeth in Ar Rass is recommended based on the knowledge gleaned from this study's findings. More accurate data could be obtained using professional double or ring flash cameras.

## Conclusions

Within the study's limitations, the actual correlation between maxillary teeth width as perceived from the front view is unidentified with 33 participants (52%). Golden percentage with 16 participants (25%) and modified golden percentage with 10 participants (16%) ranked second and third, respectively, whereas, amongst perceived maxillary anterior teeth on natural dentition, Preston’s proportion did not exist. Based on our study, a new formula for calculating the relation between maxillary anterior teeth width could be raised and should be tested on a wider population.
